# Predictive modeling of pH on the transport of Co(II) Ions from aqueous solutions through supported ceramic polymer membrane

**DOI:** 10.1038/s41598-024-63854-7

**Published:** 2024-06-26

**Authors:** A. T. Kassem, M. M. S. Ali, N. M. Sami

**Affiliations:** Hot Laboratories and Waste Management Center, Egyptian Atomic Energy Authority, Cairo, 13759 Egypt

**Keywords:** Supported ceramic polymer membranes, Cobalt (II), D_2_EHPA, Predictive model, C_4_H_10_N_2_ strip, Chemistry, Materials science

## Abstract

Optimal pH is essential for efficient cobalt extraction from polymeric membrane systems, with D_2_EHPA used as an extractant for Co(II) at pH < 7, achieving 47% efficiency. The pH of piperazine as a stripping agent increases to a concentration of 0.48 M, and the extraction efficiency of Co(II) > 80%. Depending on the functional group of (C_4_H_10_N_2_), the optimal pH for separation was 9.8. The study revealed that pKa value was calculated to predict the ideal pH, and its value was 9.73, which is nearly to the pH, since the pH of the strip concentration and the properties of the membrane affect the extraction of cobalt at 30 ^°^C. The partition ratio indicates the high distribution of the extract in supported ceramic polymer membrane (SCPM). The ceramic component provides mechanical strength and rigidity to the overall membrane structure, allowing it to withstand high pressures and temperatures during operation Study various factors such as the effect of pH on the ionization of the extract; effect of pH on band ionization; effect of pH on the temperature in the extract, effect of pH on the solute, effect of the band at different pH ranges and a comparison was made between the predictive model and experimental data that was proven through mathematical modeling using the MATLAB program.

## Introduction

These membranes are designed to address the weaknesses associated with traditional polymeric or ceramic membranes by utilizing the unique property characteristics of each material^1,2^. Ceramic-polymer supported membranes are a genre of advanced membrane materials that bring together the strengths of ceramic and polymer components to achieve enhanced performance and versatility. This hybrid architecture bestows enhanced mechanical strength, thermal stability, and chemical resistance with added benefits in terms of fouling mitigation, thus making these membranes very attractive for use in the most demanding industrial and environmental applications. More research and development efforts in the area of ceramic-polymer-supported membranes are underway to develop optimal material compositions, structures, and manufacturing processes for even higher performance, efficiency, and cost-effectiveness. As these membranes continue to evolve, they are likely to play a greater role in diverse technologies of separation and purification across a large number of industries. The study aims to develop a reliable model for predicting the behavior of Co(II) ions in support ceramic polymer membranes under different pH conditions. Co(II) ions can be found in various forms, such as free metal ions, hydroxide complexes, or ligament complexes. The charge of a membrane depends on the pKa values of its functional groups and the presence of pH-responsive species^3,4^. Common functional groups in membranes include carboxyl (COOH) and amino (NH_2_) groups. The pKa values vary depending on the pH, with carboxyl groups having a positive charge and amino groups having a negative charge^5^. Co(II) ions can be found in various forms, such as free metal ions, hydroxide complexes, or ligament complexes^6–9^. Common functional groups in membranes include carboxyl (COOH) and amino (NH_2_) groups. To develop a reliable model for predicting the behavior of Co(II) ions in support ceramic polymer membranes under different pH conditions. The pKa values vary depending on the pH, with carboxyl groups having a positive charge and amino groups having a negative charge^10–18^. The presence of competing metal ions or complexities can affect the binding and transporting of Co ions through the membrane. The size and structure of the membrane pores where morphology, the size of the pores and the structure of the supporting ceramic polymer membrane also play an important role in the transfer of ions Co(II). The pore size selectively determines the membrane size, allowing or restricting the passage of Co(II) ions based on their moisturizing size. In addition, membrane structure and zigzag affect prevalence rates and transport pathways of Co ions. Where, pH variations can affect membrane swelling or contraction, changing transport characteristics and selectivity towards Co ions. pH levels have a strong impact on ion transfer mechanisms such as diffusion, complexity and electrostatic reactions between the membrane and cobalt ions. Collect a comprehensive dataset of previous experimental measurements on the transfer of cobalt ions through ceramic-supported polymer membranes with different pH values^19^. A benefit is that ceramic materials can enhance the system's extraction efficiency and capacity. Further, the use of ceramic adsorbents is also going to make the overall extraction process easier and more^20^. The design of the ceramic polymer membrane is to provide explicit pore sizes and specific surface properties such that it enhances the diffusion and partitioning of Co(II) ions through the interface across the membrane^21,22^. Which will lead to increasing extraction efficiency and capacity over the classical methods of solvent extraction^23^. The design of the ceramic polymer membrane is to provide explicit pore sizes and specific surface properties such that it enhances the diffusion and partitioning of Co(II) ions through the interface across the membrane. Lastly, selective transfer: the ceramic polymer membrane can be designed to selectively transfer the target solute from the aqueous phase to the organic phase to improve the separation factors and purity of the extracted components^24,25^. The dataset should have included information such as pH, initial Co ion concentration, transport time and concentration of cobalt ions. It identified relevant variables that are likely to affect transport rates or concentrations of Co(II) ^6,8,26–29^. When using the predictive model by adapting the selected features to the transport rates or concentrations of the observed Co(II) ions.

The concentration of Co(II) ions observed in aqueous streams can vary significantly depending on the source and nature of the water. The specific design and operating parameters of the supported ceramic polymer membrane, such as the pore size, membrane material, and extractant selection, can be optimized to maximize the removal and recovery of Co(II) ions from the aqueous streams, making this technology a valuable tool for the sustainable management of cobalt-containing waste and the recovery of this important metal In industrial effluents or wastewater streams, the concentration of Co(II) ions can range from a few milligrams per liter (mg/L) to several hundred mg/L, depending on the specific industrial processes and the level of contamination^12^ In natural water bodies, such as rivers, lakes, or groundwater, the concentration of Co(II) ions is typically much lower, often in the range of a few micrograms per liter (μg/L) to a few hundred μg/L, due to the natural geochemical cycling and dilution effects^29^. The removal of Co(II) ions from aqueous streams is essential to mitigate the potential environmental and health impacts of cobalt contamination^17^. But previous models can help improve membrane-based filtration processes to improve cobalt removal^25^. Cobalt ion levels have a strong effect on ion transfer mechanisms such as diffusion, complexity and electrostatic interactions between membranes^30–32^. The evaluation of previous predictive models for this purpose had no impact on the study of mass transfer through the study of the stripping agent for the transfer of elements such as cobalt ions (II) ^4,26,27^., the effect of pH on the transport of the elements through stripping solution and providing a valuable tool for designing and improving separations involving Co(II) ions in aquatic solutions^33–39^ In the report Mixed Matrix Polyamide Membranes (MMPAMs) have been used in water treatment applications, especially in reverse osmosis (RO) and nanofiltration (NF) processes to remove cobalt from contaminated water. These membranes are known for their excellent separation performance and high rejection of solutes and contaminants. This, in addition to its combination with SiO_2_ silica as filler material, has many advantages such as enhanced mechanical strength and stability of the polyamide film, which reduces the risk of physical damage or contamination during operation. Second, the filler can provide additional adsorption sites or surface functionalization, leading to improved solute rejection and selectivity. Moreover, the filler can modify the membrane surface properties, such as hydrophobicity or hydrophobicity in Fig. S1. Thus affecting the membrane fouling resistance and flow properties.

## Theory

The effect of pH on various processes, several equations play an important role in this work; The pH of a solution is determined by the concentration of hydrogen ions [H^+^] present. It is calculated using-log [H^+^]. Acid dissociation constant K_a_ acts as the equilibrium constant for dissociation of an acid in aqueous phase, such as H_2_O. It quantifies the extent to which an acid donates protons [H^+^] in an aqueous solution according Eq. (1) ^39–41^.

1 $$ K_{a} = \left[ {H^{ + } A^{ - } } \right]/\left[ {HA} \right]$$

where [H^+^] and [A^−^] acts hydrogen ion concentration, and concentration of the conjugate base where [HA] is the concentration of the acid. According to Eq. (2)^42^, Henderson–Hasselbalch equation, it is describing the relates the pH of a solution to the dissociation constant pk_a_ and the ratio of the concentrations of the conjugate base [A]^−^ to the acid [HA] and describe acid–base equilibria

2 $$ pH = pK_{a} +\log \left[ {A^{ - } } \right]/ {\left[ {HA} \right]} .$$

For the Nernst, Eq. (3)^43^ is used to calculate the electrode potential of a half-cell in an electrochemical system, where E^*^ standard potential, R gas constant (8.314 J/mol k), T is the temperature (kelvin), n charge number of the ion species involved, F Faraday's constant (96.485 C/mol), Q is the reaction quotient. And concentrations (activities) of the oxidized and reduced species according equation:

3 $$ E = E^{*} - \left( {{\raise0.7ex\hbox{${RT}$} \!\mathord{\left/ {\vphantom {{RT} {nF}}}\right.\kern-0pt} \!\lower0.7ex\hbox{${nF}$}}} \right)^{*} \ln Q $$

In addition to the Maxwell-Oken equation, there are other equations and models that can be used to describe ceramic and polymer films with SiO_2_ fillers. Such as Brueggemann's Effective Medium Theory (EMT): Brueggemann's effective medium theory is a widely used model for predicting the effective properties of composite materials. It relates the effective property of a compound to its volume fractions and properties of its components. For electrical conductivity, the Eq. (4)^44^ can be given as follows:

4 $$ \sigma_{eff} = \sigma_{{c^{*} }} \left( {\phi - \phi_{c} } \right)^{t} $$

where σ_eff_ is the effective conductivity, σ_c_ is a critical conductivity, φ is the volume fraction of the filler phase, φ_c_ is the percolation threshold, and t is the critical exponent. The Maxwell–Stefan model is a multicomponent diffusion model that describes the transport of different species in a mixture. This allows the analysis of the diffusion behavior of ceramic polymer membranes with SiO_2_ fillers. The model assumes that the diffusion flux of each component is affected by concentration gradients and interactions with other species^45^^.^ The Maxwell–Stefan equation for the diffusion flux (J_i_) of the i_th_ component in a mixture is given by the Eq. (5)^46–48^:

5 $$ J_{i} = D_{i} \nabla C_{i} \sum {\left( {D_{i} C_{i} \nabla \ln \left( {\gamma_{i} } \right)} \right)} $$

Where D_i_ is the diffusion coefficient of the i_th_ component, C_i_ is the concentration of the i_th_ component, ∇C_i_ is the concentration gradient of the ith component, γi is the activity coefficient of the i_th_ component, and the ∑ is taken over all the components in the mixture. The Maxwell–Stefan model based on ceramic and polymer films using SiO_2_ fillers may involve determining the diffusion coefficients, concentration gradients, and activity coefficients of the different components Therefore, it provides a theoretical framework but may require experimental validation and calibration to accurately predict certain ceramic polymer membrane systems. The first term on the right represents the contribution of the concentration gradient to the diffusive flux, while the second term illustrates the effect of the activity coefficient, which captures the interaction between different species^49^.

## Experimental

Preparation and characterization of a polyamide polymer membrane as a mixed matrix membrane (mmm) combined with silica (SiO_2_) filler: ‘Materials and Equipment’s. Using Polymer: m-phenylenediamine, trimesoyl chloride (0.2 mol) [purchased from Sigma-Aldrich Co. (St. Louis, MO, USA)]. For Instrument pH values of different solutions were measured by using pH meter type HANNA (USA). All samples in this report were weighed by using an analytical balance produced by Bosh (Germany). Cell supported membrane system shown in Fig. S2. Scheme for pertraction apparatus. (1) Double shield glass outer vessel; (2)Teflon cross-stirring blade; (3). Supported membrane(cellulose nitrate); (4)feed compartment; (5) strip compartment^49^. Spectrophotometric Measurements of Cobalt (II) in the HNO_3_ (was determined spectrophotometrically using UV–visible spectrophotometer (a Shimadzu UV-160, Japan).

### Procedures

The Membrane Preparation: The Membrane material: nano silicon. Membrane formation by sintering method; Membrane thickness: 100 μm. Membrane pore size as 0.1 m, particle size75 μm, and size of distribution of 0.3 as for nanofiltration and the membrane installation: as disc membrane active area: 10^2^ m^2^ and the membrane housing material as polymer for the membrane conditioning; as the membrane compaction pressure: 1 bar for the membrane conditioning time: 24 h. From previous works, a study was conducted on the concentration of cobalt ions in various extraction processes, The Table 1: Comparative Analysis of Cobalt Extraction Methods: Evaluating Concentrations and Extraction Efficiency; with a focus on aqueous metal extraction and solvent extraction methods. The membrane permeate flux stabilization: 1 × 10^−7^ m/s variation and the Performance Evaluation in the feed solution composition: acts salts and organic compounds, the pH = 5.6 and the temperature 25–30 °C. Solute rejection more than 90% for cleaning and renewal using using rotating brushes to remove accumulated foulants from the membrane surface^57,58^^.^, for 2 h or cleaning duration and the cleaning frequency weekly; the membrane replacement lifetime as 1 months. This study focuses on the preparation of ceramic polymer membranes (CPMs) using a sol–gel method. The process involves dissolving silica alkoxide in a solvent, adding it to a polymer solution, sonicating, degassing, casting, and immersing the cast film in deionized water. The membrane is then formed using m-phenylenediamine and trimesoyl chloride in N,N-dimethylformamide. The membrane is then treated with annealing temperature at 180^0^C to enhance compatibility with the polyamide polymer matrix. The study also discusses the use of –NH_3_ as functionalization techniques. Table 2 Describe types of membranes, materials and advantages, disadvantages^54–58^.

**Table 1:** Comparative analysis of cobalt extraction methods: evaluating concentrations and extraction efficiency.

Concentration of Co(II),g/L	Techniques	Extraction%	References
50	precipitate using organic acids	86.8	Hussaini et al.^50^
12	Solvent extraction	90	Zantea et al.^51^
0.73 g/L	electrochemical leaching process	98.5	Strauss et al.^52^
0.1–10	Liquid–liquid extraction	Up to 95%	Riano et al.^53^
28	supported ceramic polymer membrane	98.6%	In this work

**Table 2:** Describe types of membranes, materials and advantages, disadvantages.

Types of membrane	Membrane material	Advantages and disadvantages
Size exclusion A-Size exclusion chromatography(SEC)	Polymer molecules	Size based separation allow for characterization of the MWT distribution(chemical sensitivity) used to study the size exclusion and adsorption mechanism^54,55^
B-membrane distillation	Polymeric/ceramic PTEE,PVDF	Thermal driven separation process used both polymeric and ceramic membranes -hydrophobic type Advantage:lower thermal conductivity compared to ceramic membranes which can improve the energy efficiency of the membrane distill process -higher thermal and chemical stability -more rigid and stable structure^56^
Membrane bioreactor	Polymeric/ceramic PVDF,PE and PAN	-used for treatment of wastewater -lower cost and more flexible in their manufacturing and module Configuration compared to ceramic membrane -porous structure of polymer membrane in MBRs allows for the effective separation of biomass and treated water. Can be susceptible to fouling and scaling due to accumulation of organic matter. and this type made from material like, alumina,zirconia or titania,ceramic membrane,generally have a higher permeate flux and can with stand more aggressive cleaning conditions such strong oxidizing agents^57,58^

PVDF: polyvinylidene;PE: polyethylene; PAN: polyacrylonitrile and MBRs: membrane bioreactors.

Table 3 Transporters and aqueous systems used in Support Ceramic Polymer membranes (SCPMs) for cobalt ion transport. This step involves modification of the filler surface (FS) and can be introduced onto the FS through the chemical reaction. Nano silica particles in a suitable DMF are good solvating power for polyamide, from stable suspension using mechanical mixing and ultra-sonication to use a uniform dispersion^59^.

**Table 3:** Chemical composition transporters and aqueous systems used in Support Ceramic Polymer membranes*.*

Chemical composition of Nano-silica selectivity for ceramic supported membrane								
SiO_2_	MgO	CaO	SO_3_	Fe_2_O_3_	Al_2_O_3_	Na_2_O	K_2_O	LOI
92.8	0.11	0.079	0.28	0.032	0.09	0.93	0.019	5.66
Selected transporters and aqueous systems used in Support Ceramic Polymer membranes (SiO_2_ with Piperazine ionization)								
High mechanical strength	Chemical stability	transporters	Ionophores	Ligands	pH	Moisture	T^0^C	FG
75 MPa	SiO_2_ (strong oxidizing agents)	D_2_EHPA	Cationic	Covalent bonds (Si–OH)	> 7 < 12	Hydrophilic (good moisture stability)	1500	Two nitrogen atoms in the ring structure

FG as a functionalized group.

**Figure 1:**
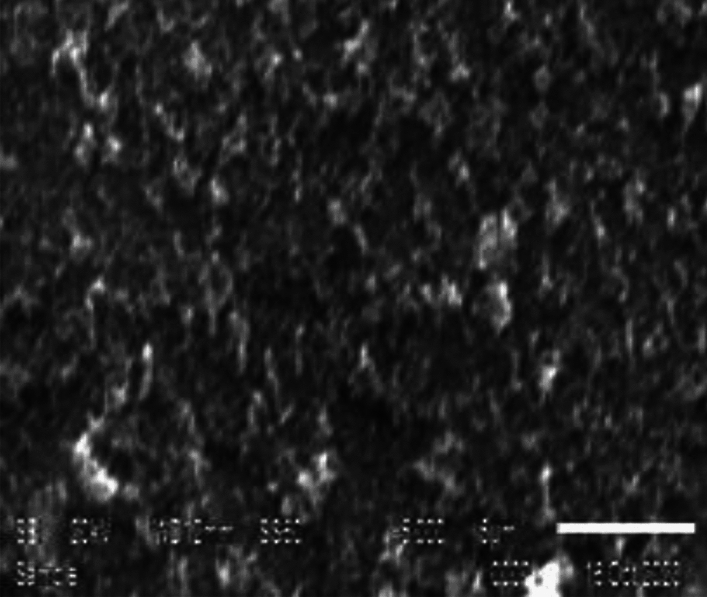
Polyamide coated silica nanoparticles Quantities of 75 gm, 150 mL DMF.

**Figure 2:**
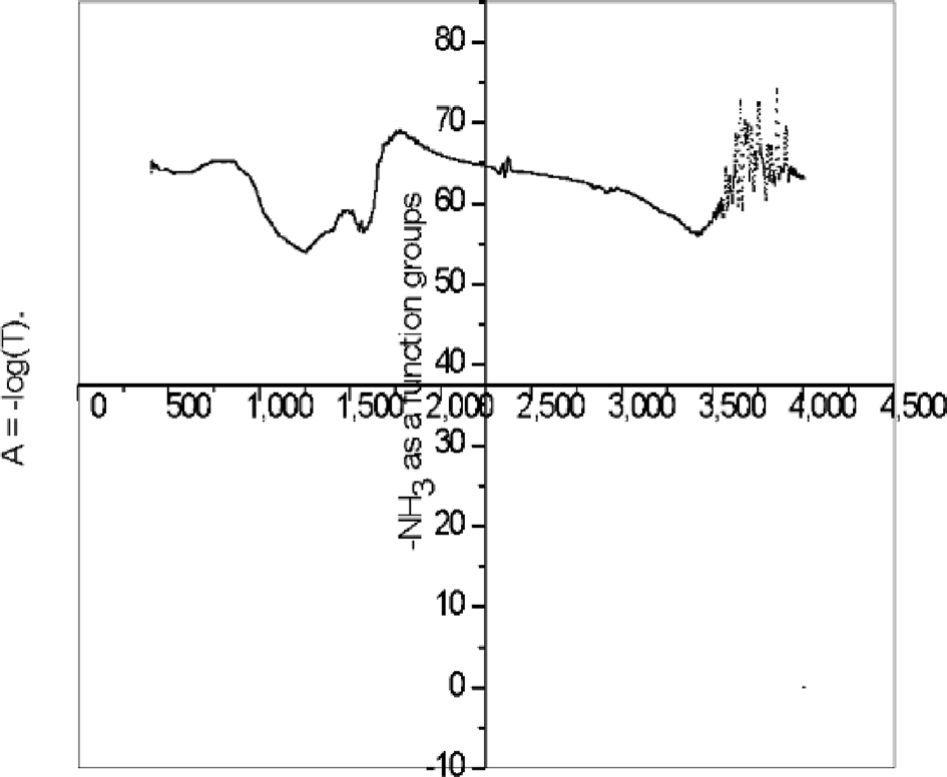
Principal of (PCA) to demonstrate data variability. The arrows demonstrate which variables are most linked with the principal components. The correlation between Absorbance and intensity for nanosilicate%.

Characterization by Fig. 1 represents the scanning electron micrographs (SEM) of Silica particles coated with polyamide. Quantities 75 g, 150 mL DMF The study uses scanning electron microscopy (SEM) to analyze polyamide-coated silica nanoparticles. The results show well-defined spherical nanoparticles coated by a thin, uniform polyamide layer. The particle size distribution is expected to be narrow, reflecting uniform coating. Surface topography shows the degree of agglomeration or dispersion of nanoparticles. Compositional analysis provides the possibility of obtaining the morphological, structural and compositional characteristics of the resulting nanomaterial. The average particle size is 75 nm, with a size distribution of 0.3 Fig. 2. Principal components analysis (PCA) To demonstrate the variance of the data for modification the crystallinity ratio can be calculated. Principal component analysis (PCA) is used to analyze data variability and crystallinity of ceramic-supported membranes. Principal components analysis (PCA) is a statistical method that reduces the dimensional of complex data sets by transforming original variables into principal components (PCs). It helps identify patterns and relationships in data, such as the relationship between ion absorption and nano sized silicate in ceramic polymer membranes. PCA calculations and a scatter plot matrix are used to create a relationship between variables and main components of the ceramic membrane, determining the percentage of nano-silica in the preparation. According in Eqs. ^60^.

6 $$ {\text{Total variance}} = \sum\limits_{i = 1}^{n} {\lambda_{i} } $$

where λ_i_ as amount of variance by each component; n = data set (Total variance)*.*

7 $$ VarianceExplained_{i} = \frac{{\lambda_{i} }}{{\text{Total variance}}}$$

The ratio of variance (EVRi) for each component.

8 $$ EVRi = \frac{{\lambda_{i} }}{{\sum\nolimits_{i = 1}^{n} {\lambda_{i} } }} $$

(CEVR_i_) or cumulative explained variance ratio.

9 $$ CEVR_{k} = \sum\limits_{i = 1}^{k} {EVR_{i} } $$

**Figure 3:**
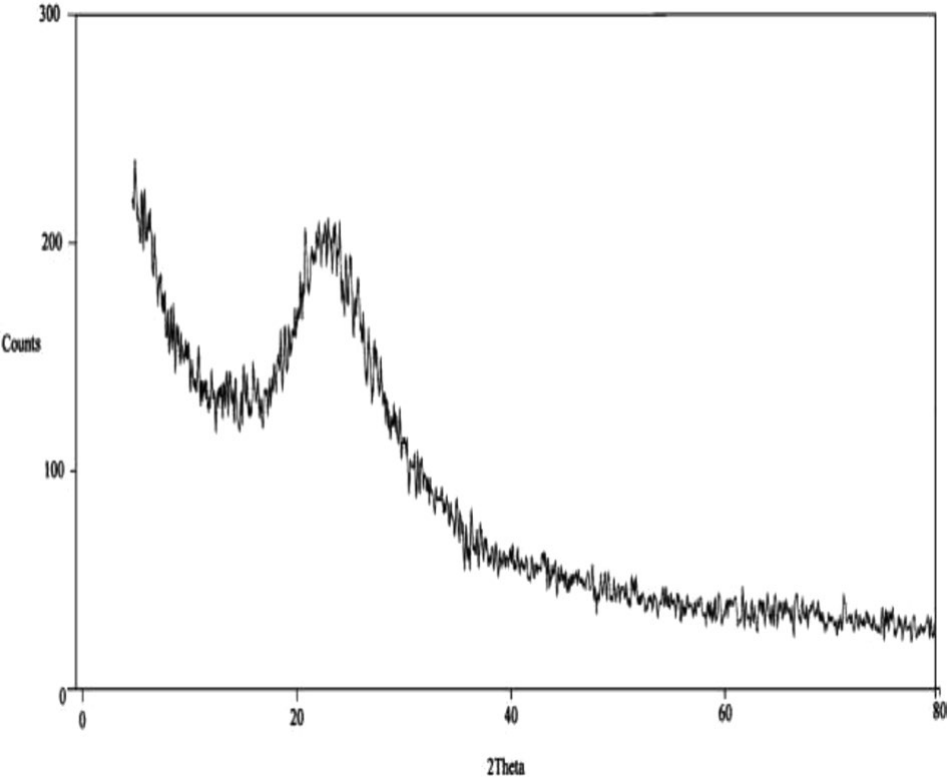
XRD pattern of nano silica.

The value of CEVR_k_ principal components; n = total number of principle components, λ_i_ eigenvalue corresponding to the i_th. Total variance of Ʃλi Variance Explained_i_ or the amount of variance by the i_th and EVR_i_ ratio for the i_th principal components. And CEVR_k_ cumulative explained variance ratio after considering the first k principal components. Data are collected from X-ray diffraction (XRD) patterns, SEM images, and other measurements. PCA is applied to identify the main components that contribute to the variation in membrane properties. Crystallinity is calculated and its relationship to film crystallinity is determined. This information can guide improvements to achieve desired properties. The application requires the best materials and analytical techniques. Figure 3 shows XRD pattern of nano-silica^60^.

10 $$ \% crystallinity = \frac{{\rho_{c} \left( {\rho_{s} - \rho_{\alpha } } \right)}}{{\rho_{s} \left( {\rho_{c} - \rho_{\alpha } } \right)}}\times100 $$

where ρ_c_ and ρ_ɑ_ measure by experimentally; for XRD possible for crystalline phase ρ_c_ density of 100% crystalline phase; ρ_a_ density of wholly amorphous phase and ρ_s_ as amorphous polymer crystalline at 23.65% of polymer chains exhibit a more ordered or crystalline arrangement, while 76.35% as remaining in an amorphous disordered at pH 9.8 and pk_a_ 12.004.

### Effect of temperature on ceramic membrane

The ceramic polymer supported membrane filtration system, i.e. To control temperatures: An electrical circuit is used to control temperatures during the experiment as shown in Fig. S3, and to maintain the required temperatures. And analyze the collected data to evaluate the effect of temperature on cobalt separation efficiency**.**

### Piperazine ionization

The chemical equation for C_4_H_10_N_2_ ionization states that it reacts with excess of (OH^−^) in an alkaline environment to form its conjugate base, the piperazine ion. This process is negatively charged and stabilized by resonance within its ring structure. The degree of ionization and equilibrium concentration depend on the pK_a_ which nealy from pH in this report, which is a measure of its acid/base content. The chemical equation representing the ionization of piperazine can be written as follows:

$$ {\text{C}}_{{4}} {\text{H}}_{{{1}0}} {\text{N}}_{{2}} + {\text{ OH}} - \rightleftharpoons {\text{C}}_{{4}} {\text{H}}_{{9}} {\text{N}}_{{2}} - \, + {\text{ H}}_{{2}} {\text{O}}\;\;\;\;{\text{R}}_{{1}} $$

## Results and discussions

### Interaction mechanisms for the effect of Piperazine (C_4_H_10_N_2_) on the Co(II) ions

When piperazine undergoes ionization at a pH > 7, it can interact with Co(II) ions in solution. The piperazine ion (C_4_H_9_N_2_^−^) can coordinate with the Co(II) ion, forming a complex through a coordination bond. The specific complex formed between piperazine and Co(II) will depend on the stoichiometry and the coordination geometry of the complex^61–63^. represent the interaction mechanisms between piperazine (C_4_H_10_N_2_) and Co(II) ions in a ceramic polymer membrane through chemical equations: In complexation: (R_2_)

$$\text{Co}^{2 + } + \text{C}_{4} \text{H}_{10} N_{2} \Leftrightarrow \left[ {\text{Co}\left( {\text{C}_{4} \text{H}_{10} \text{N}_{2} } \right)} \right]_{2}\,\,\,\,\text{R}_2\text{ complexation.}$$

The formation of this complex can enhance the solubility of cobalt and its transport across the membrane, which may lead to improved extraction efficiency (%EE). Reaction of D_2_EHPA with Co(II) ions: D_2_EHPA is an extractant widely used to recover metal ions, including Co(II), in ceramic supported membrane processes. The extraction mechanism usually involves the formation of a neutral dissolved mineral complex D_2_EHPA:

$$\text{Co}^{2 + } + 2\text{(HR)}_{2} \Leftrightarrow\left[\text{Co}(\text{HR})_{2}  )\cdot 2\text{HR}\right]\,\,\,\,\text{R}_3\text{ Surface adsorption}$$

Where HR represents the D_2_EHPA molecule. The formation of this complex allows cobalt to be selectively transferred from the aqueous phase to the organic phase, facilitating the separation and purification of cobalt. Synergistic Effects: In the presence of both piperazine and D_2_EHPA, there may be potential synergistic effects on cobalt extraction and separation. The piperazine-cobalt complex can react with D_2_EHPA in the organic phase, leading to the formation of a ternary complex:

$$ \left[ {\text{Co}\left( {\text{C}_{4} \text{H}_{10} \text{N}_{2} } \right)} \right]_{2} + 2\text{(HR)}_2 \leftrightharpoons [\text{Co}(\text{C}_4\text{H}_{10}\text{N}_2)\text{(HR)}_2]+2\text{H}^+ \,\,\,\,\text{R}_4\text{  Ion exchange}$$

This ternary complex can exhibit improved extraction efficiency, selectivity, and stability compared to single extraction mechanisms. The interaction between piperazine formulation and D_2_EHPA extraction should be carefully studied to optimize the overall solvent extraction process for cobalt recovery^64^.

The formation of the piperazine-Co(II) complex can be represented by the following Reaction (R_5_):

$$[\text{C}_4\text{H}_9\text{N}_2]^-+\text{Co(II)}]\leftrightharpoons\text{Co}^{3+}+\text{ (piperazine) }x+e^- \,\,\,\,\text{R}_5\text{  Redox Reactions}$$

Ion exchange involves the exchange of Co(II) ions with piperazine molecules within the membrane. The complexation reaction forms coordination complexes between piperazine and Co(II) ions. In this equations, the piperazine ion (C_4_H_9_N_2_^−^) coordinates with the Co(II) ion to form the piperazine-Co(II) complex, represented as Co(piperazine) x. Which can vary depending on the molar ratio of piperazine to Co(II). The formation of the complex between piperazine and Co(II) can impact the solubility, stability, and reactivity of Co(II) ions. It can also influence the extraction and separation of Co(II) from an aqueous phase into an organic phase, as piperazine acts as a carrier for the metal ion. The properties of the piperazine-Co(II) complex will depend on factors such as the pH at 9–12, concentration at 28,000 mg/L, and the function group of strip or competing species^61^. To estimate the concentration of the feed phase in the ceramic polymer membrane, we can use Fick’s Law of Diffusion can be calculations: The permeability coefficient of the ceramic polymer membrane for cobalt ions is 1 × 10^–7^ m/s at 303.15 K or 30 °C. according Fick’s low of diffusion^62^.

11 $$ J = - P\frac{dC}{{dt}} $$

where J = flux of cobalt(II) through the membrane (mol/m^2^s); P = permeability coefficient of membrane m/s and dc/dt concentration gradient via the membrane (mol/m^3^). in this work J = 3.2 × 10^–10^(mol/m^2^s). and the flux equal.

12 $$ J = - P\frac{{C_{0} - C_{f} }}{L} $$

where: C_f_ as concentration in the feed phase of cobalt ions (mg/L), L = 0.1 or membrane thickness(m) at equilibrium J = 0. Concentration of feed phase of cobalt ions act:

13 $$ C_{f} = C_{0} - \frac{JxL}{P} $$

at equilibrium, the concentration of cobalt ions in the feed phase of the ceramic polymer, C_0_ = 28 mg/L and C_f=_28.000 mg/L.

$$MxAy(s) \Leftrightarrow xM^{z + } \left( {aq} \right) + yA^{a - } \left( {aq} \right) \,\,\,(R_6)$$

### Effect of pH on the ionization of the D_2_EHPA as extractant

**Figure 4:**
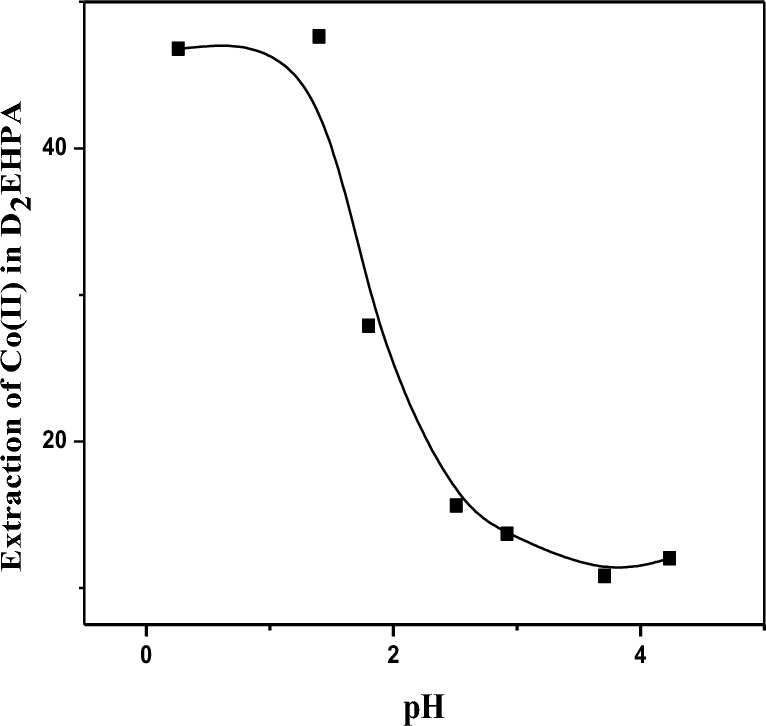
Effect of pH on the ionization of the D_2_EHPA as extractant of cobalt ion (II) = 28 g/L at pH 6.8 on the extractant using [(C_4_H_10_N_2_)] ~ 0.48 M, [D_2_EHPA] ~ 3.3 mol/L, [HNO_3_] ~ 2. 5 mol/L at pH = [0–5].

The effect of D_2_EHPA at a concentration of 3.3 mol/L on the extraction of Co(II) at a lower pH < 7 in the SCPM system is shown in Fig. 4. The D_2_EHPA extraction percentage (%E) was lower than in the strip case, which means the appearance of positively charged species (D_2_EHPA –H^+^). This protonated form has a higher affinity for Co(II). Therefore, at lower pH values the efficiency of Co(II) extraction by extractant can increase due to increased H^+^ as the extractant and Co(II) ion are enhanced. Depending on the pH that is completely dependent on the extraction. In the pH range from 0 to 5, the %E_e_ decreases as the pH decreases, and this is clear in the figure. We find that the specific behavior and performance of D_2_EHPA at a Co(II) extraction ratio of Co(II) = 47.4% depend on the D_2_EHPA concentration, pH of the solution, membrane properties, and the presence of other competing ions^63,64^.

$$ MxAy(s) \Leftrightarrow xM^{z + } \left( {aq} \right) + yA^{a - } \left( {aq} \right) $$

where M^z+^and A^−a^ are the Co^2+^ of the salt respectively; where the solubility of the salt depends on the pH of the solution, it can affect the protonation.

14 $$ MxAy\left( s \right) \Leftrightarrow M^{z + } \left( {aq} \right) + yA^{a - } \left( {aq} \right)A^{a - } \left( {aq} \right) + H^{ + } + \left( {aq} \right) \Leftrightarrow HA\left( {aq} \right) $$

This Eq. (14) represent equilibrium position and therefore the solubility of salt will depends on the pH of the solution.

### Effect of pH on stripping and ionization phase

**Figure 5:**
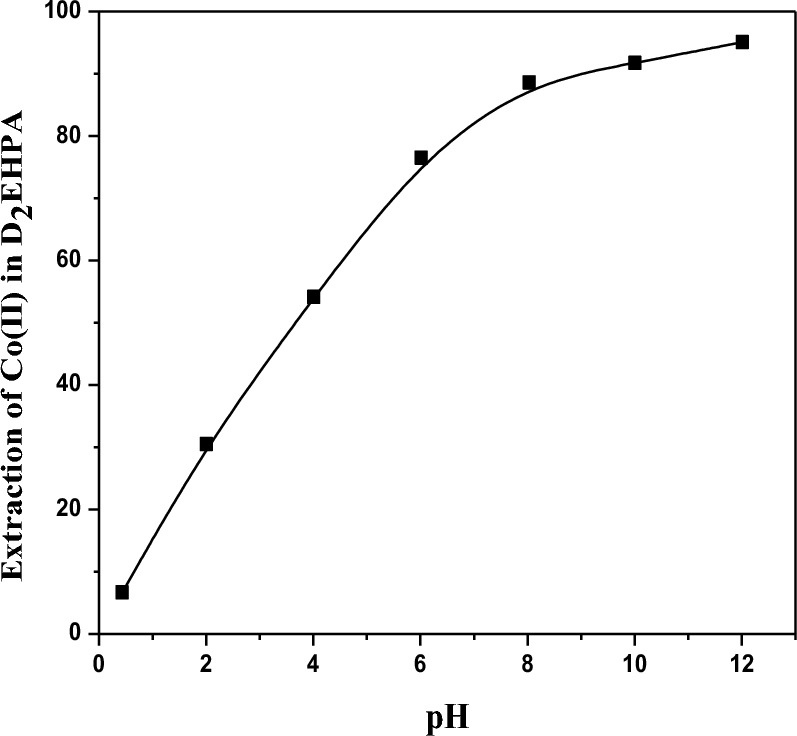
Effect of pH on stripping and ionization phase of the Co(II) % E from [(C_4_H_10_N_2_)] ~ 0.48 M, [HNO_3_] = 2.5 mol/L at [Co^2+^] = 28 g/L mol/L, [HNO_3_] ~ 2.5 mol/L at pH [9.8]. [D_2_EHPA] ~ 3.3 mol/L.

The pH of the solution affects the behavior of piperazine due to the presence of Co(II) ions and ceramic polymer films (CPFs). pH plays an important role in the ionization state of strip and its reaction with Co(II) ions. Figure 5; the effect of pH on stripping in the presence of ions in a SCPM system is demonstrated: Piperazine ionization: At pH > 7, piperazine ionizes by accepting a proton from the hydroxide ion in solution. This ionization leads to the formation of strip ions (C_4_H_9_N_2_)^−^, which are −ve ions, which react with ions in the solution, forming complexes. The specific complex formed depends on stoichiometry. The pH of an aqueous solution greatly affects the stripping and ionization of mineral complexes, especially those containing negatively charged ions. Stripping efficiency is enhanced at lower pH values, with increasing hydrogen ion concentration, resulting in protonation of negatively charged D_2_EHPA metal complexes. The anionic form of D_2_EHPA is more efficient at extracting metal ions, such as Co(II)^64–66^. pH-induced changes in membrane properties: The pH of the solution can also affect the properties of the membrane^67^. Changes in pH can also affect the surface charge, porosity, and permeability of the membrane, which in turn affects the transport of piperazine and ions., via the membrane. PH-dependent changes in membrane properties to improve separations for Optimizing separation or extraction processes are nearly 89.7% dependent on the function group of stripping agent.

### Effect of pH on temperature in extractant

**Figure 6:**
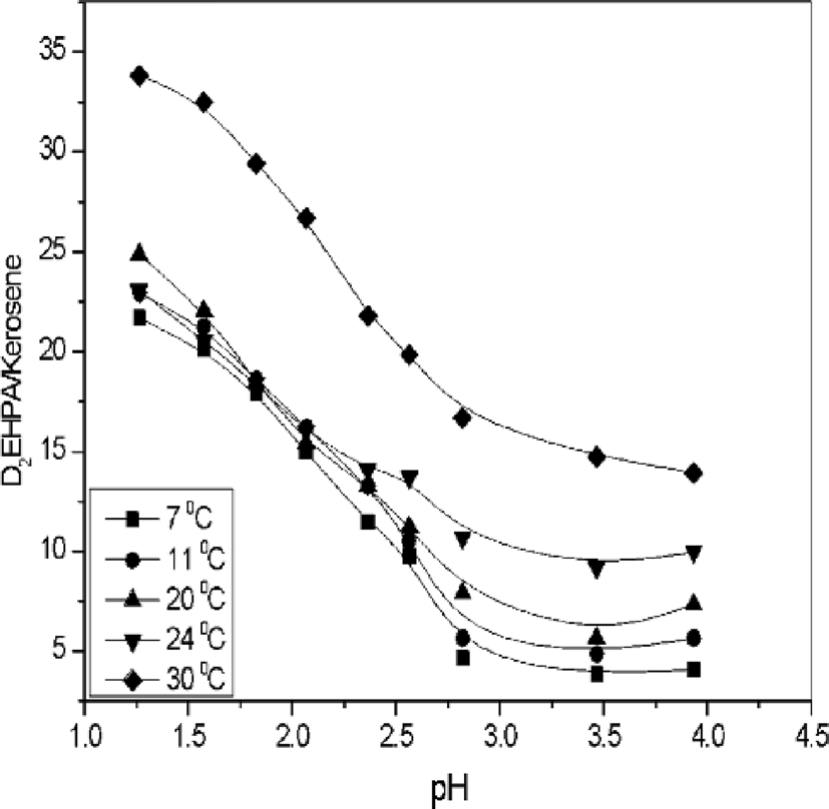
Effect of pH on the temperatures in [D_2_EHPA] ~ 3.3 mol/L, [HNO_3_] ~ 2.5 mol/L at pH = [1–4.5].

Effect of pH on temperature in D_2_EHPA (1–4.5) extraction systems. Experiments were conducted at four different temperatures while keeping other factors constant to study the effect of temperature. Specifically, the first set of experiments was performed to examine the effects of different extraction temperatures, including 7–30 °C. The temperature of the extraction system was maintained using the experimental temperature conditions described in the Experiment section. Transport experiments were performed at room temperature (20–25 °C). According to Darcy's law, the flow rate was calculated according to different parameters calculated through experiments. Affecting extraction efficiency at 34.8%. As shown in Fig. 6. At high temperatures enhance extraction kinetics by increasing molecular diffusion rates and reducing the viscosity of the organic phase^68^. Optimization of pH and temperature conditions is necessary to achieve the desired E_e_ and kinetics in D_2_EHPA-based SCPM systems and depends on the functional group of the extractant according to Eq. (15)^69^.

15 $$ E_{e} = \frac{K.D.pH}{{K.D.pH + K + 1}} $$

pH represents of the solution. D is the distribution ratio, reflecting the ratio of solute concentration in the organic phase to that in the aqueous phase. K is the equilibrium partition coefficient, indicating the relative distribution of the solute between the phases at equilibrium. E_e_ is the extraction efficiency, which accounts for the overall effectiveness of the extraction.

### Effect of pH on the temperature in C_4_H_9_N_2_

**Figure 7:**
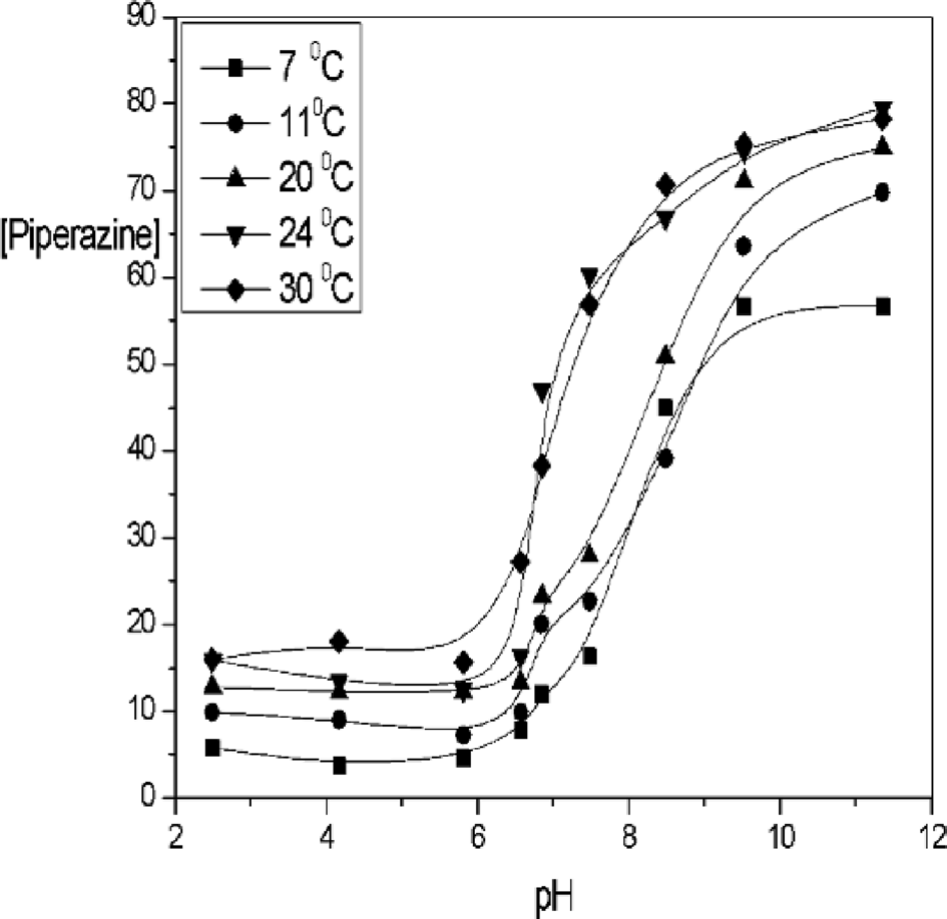
Effect of pH on the temperatures in [(C_4_H_10_N_2_)] ~ 0.48 M] =] ~ 3 × 10^–2^ mol/L, pH = [9.8].

In the HNO_3_, we find that the acid dissociates completely in water, which means that all of its molecules dissociate into ions in the solution. Therefore, the acid dissociation constant (K_a_) is slightly lower than the pH, indicating weak dissociation. When the pH of the solution is higher than K_a_. This means that all acid molecules do not ionize into hydronium ions (H_3_O^+^). In the Fig. 7. The pH > 7, while the value of ka is close to the pH. However, the situation is different in the presence of piperazine, which is known as a heterocyclic organic compound containing two nitrogen atoms in its structure. The pKa values of the nitrogen atoms in piperazine depend on the specific nitrogen atom and the conditions of the solution^57–72^. When calculating the pKa values of the nitrogen atoms in piperazine: In the hexagonal ring: the pKa of the nitrogen atom in the hexagonal ring of piperazine is about 9.73 so, that the study relied on the presence of the piperazine functional group in the hexagonal ring. Extraction yield of cobalt (II) ions using piperazine at 80.4% shown in the Reactions. (R_7_–R_10_).

$$ Co(II) + NO_{3}^{ - } + 2S_{oi} \mathop{\longrightarrow}\limits^{F - M}Co\left( {NO_{3} } \right)^{2 - } .2S_{oi} \,\,R_7$$

$$ 2H^{ + } + NO_{3}^{ - } + nS_{oi} \mathop{\longrightarrow}\limits^{F - M}2HNO_{3} .nS_{oi} \,\,R_8$$

$$ H_{2} O + mS_{oi} \mathop{\longrightarrow}\limits^{F - M}H_{2} O.mS_{oi}  \,\,R_9$$

$$ Co(II) + C_{4} H_{10} N_{2} \to Co - \left[ {C_{4} H_{10} N_{2} } \right]^{ - 2} - 2\left( {complex} \right)   \,\,R_{10}$$

where S_ol_, indicates the solvent, D_2_EHPA and the bar symbolize organic phase. Also, the ‘F-M’ indicates the ‘feed -membrane’ interface and ‘membrane-strip’ moderator, at these special interfaces the complexation and reverse reaction happens respectively. Piperazine is a strong stripping agent for formation of such a complex, in this case with the further increase in cobalt ion concentration, the complex of Co^2+^—Piperazine is formed which is insoluble in aqueous medium formation of precipitate.

$$ Co(II) + piperazine \to Co - \left[ {piperazine} \right]^{2 - } \left( {complex} \right)    \,\,R_{11}$$

$$ Co^{2 + } + Co - piperazine \to Co^{2 + } \to piperazine   \,\,R_{12} $$

**Figure 8:**
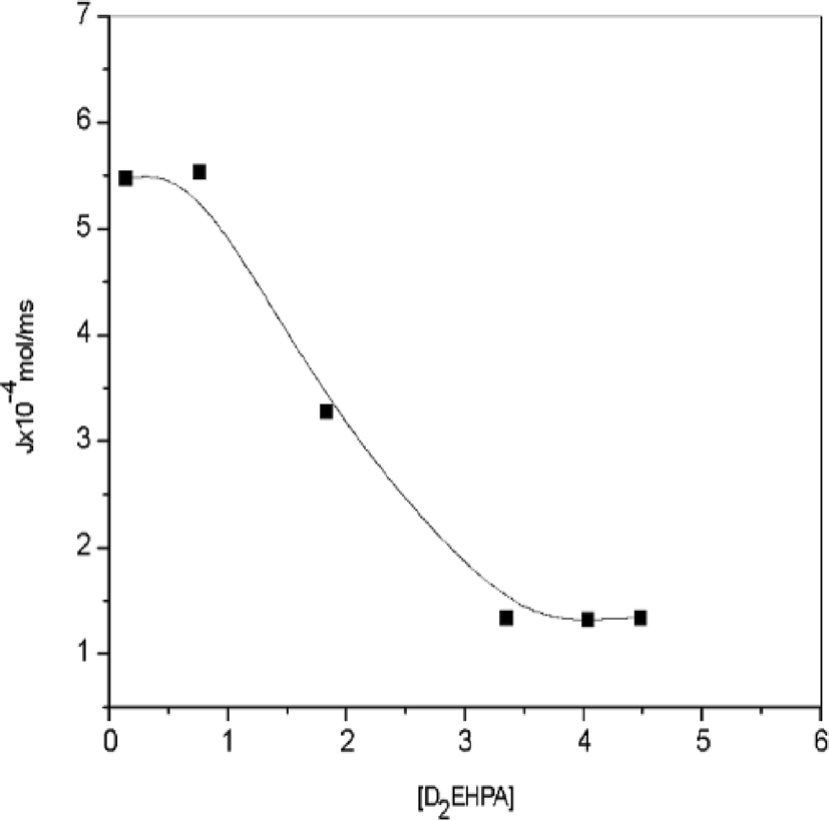
Effect of Co(II) ion concentration on the flux (J) Permeability P = 1 × 10^−7^ m/sat T = 303.15 K or 30^0^C.

The extraction may have been affected by the depositions that have accumulated in the porous ceramic membrane. The effect of Co(II) ion concentration on the flux is depicted in Fig. 8. The flux (J) shows a decreasing trend with a maximum value of 5.45 × 10^–4^ to 2.3 × 10^–4^ mol/m^2^ of Co(II). The reduction in the flux value is due to the mentioned above reason.

### Effect of pH on the solute

**Figure 9:**
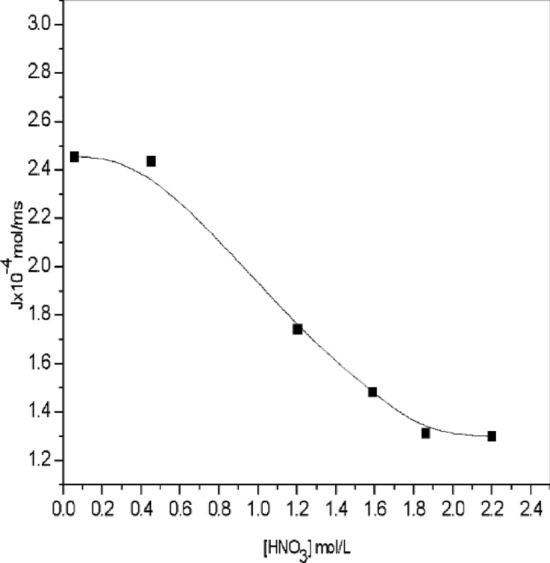
Effect of HNO_3_ concentration on flux of cobalt ion [Co^2+^] 3.4 × 10^–3^ mol/L, [Piperazine] 0.48 M, [D_2_EHPA] 3.3 mol/L and [HNO_3_] 0.5–2.2 mol/L at pH 9.8 at 30 ^°^C.

The effect of HNO_3_ concentration on cobalt ion flux depends on the specific system or experimental conditions. Higher concentrations can cause increased dissociation of H^+^ ions, affecting cobalt ion solubility and mobility. pH, affects the type of cobalt ions and availability. pH changes affect the rate of diffusion and penetration of acid into membranes, affecting the transport of cobalt ions and material separation. The ionization region, where acidic solutes like nitric acids are more easily extracted, depends on the type of phase at pH 9.8, shown in Fig. 9. According to Eq. (16)^63^, and Reaction (R_13_). pH affects the degree of ionization of the solute to be extracted at pH at 9.8, which is called the ionization region and lies between [HNO_3_] [0.5–1.3 M] slope = − 0.64, intercept = 2.68041. For acidic solutes such as nitric acids, a pH > 7 results in more solute being in its ionized form, and solutes are more easily extracted into the organic phase. The distribution ratio and equilibrium partition of the solute depend greatly on the type of phase. This shows that the effect of nitric acid is greater on the stripping phase than the extractant phase.

16 $$ \alpha = m.pH + c $$

α represents the degree of ionization of nitric acid. As the pH increases, indicating a more alkaline environment, the degree of ionization of nitric acid decreases, affecting its behavior in extraction processes c is the intercept of the linear relationship. Given the slope (m) and intercept (c) values provided, the equation becomes: ɑ =  − 0.64.pH + 2.68041. This equation captures how the degree of ionization (ɑ) of nitric acid changes with variations in pH within the specified ionization region and concentration range.

$$ \left[ {\left( {D_{2} EHPA} \right)} \right]NH^{ + } + \left[ {CoNO_{3 + n} } \right] \Leftrightarrow \left[ {D_{2} EHPA} \right]NHCONO_{3 + n} \,\,R_{13}$$

### Effect of [Piperazine] at different pH

**Figure 10:**
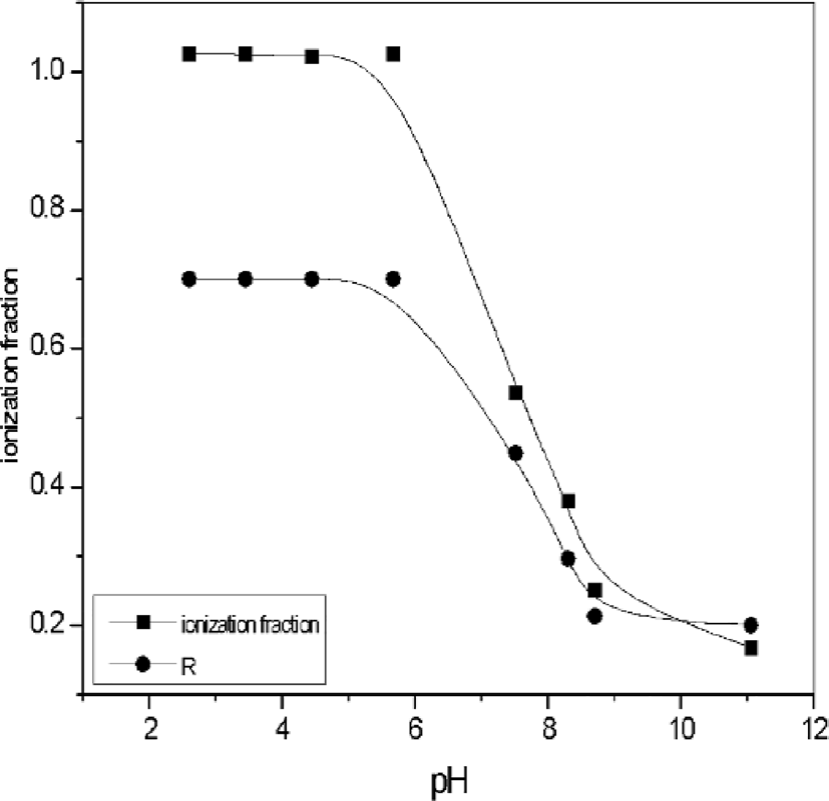
Influence of pH of Piperazine strip ionization and fraction of extraction of [Co^2+^] = 4 × 10^–3^ mol/L, [D_2_EHPA] 3.3 mol/L and [HNO_3_] 2.5 mol/L at pH [1–11].

**Figure 11:**
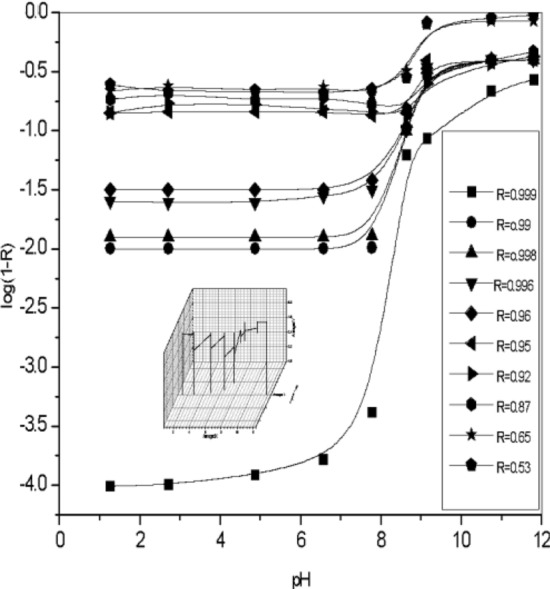
Modeling pH of Piperazine stripping phase 0.45 M on Co(II) ion concentration diffusion with CPSM with carrier D_2_EHPA in kerosene.[Co^2+^] = 3.4 × 10^–3^ mol/L, L, [D_2_EHPA] 3.3 mol/L and [HNO_3_] 2.5 mol/L at pH [0–12].

**Figure 12:**
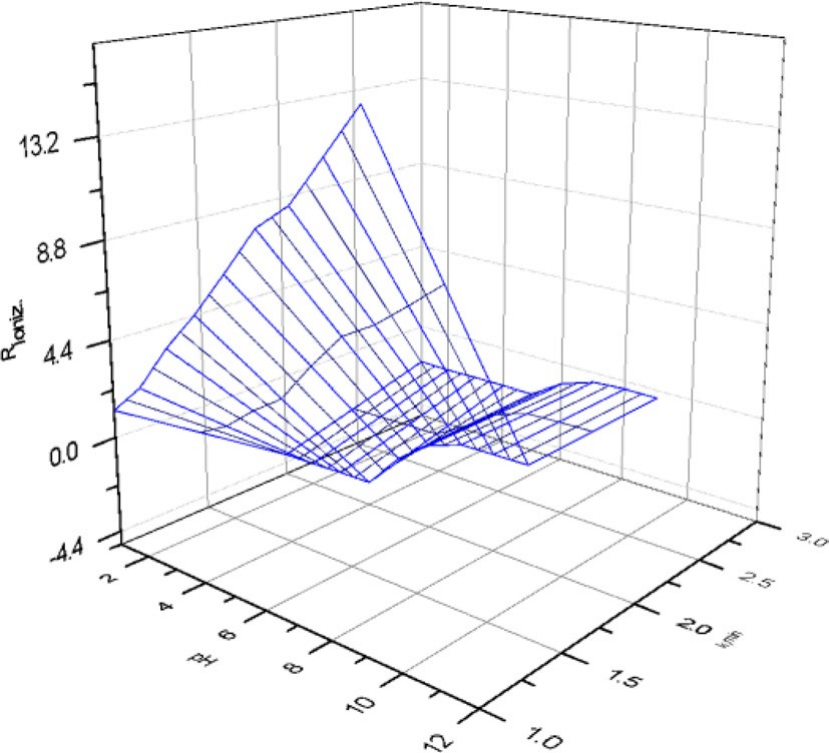
3D for effect of [piperazine] 0.45 M as stripping phase at pH [1-12] on the rate of reaction K,_min_ and the yield of diffusion of Co(II) ion concentration, [Co^2+^] = 3.4 × 10^–3^ mol/L, mol/L, [D_2_EHPA] 3.3 mol/L and [HNO_3_] 2.5 mol/L.

**Figure 13:**
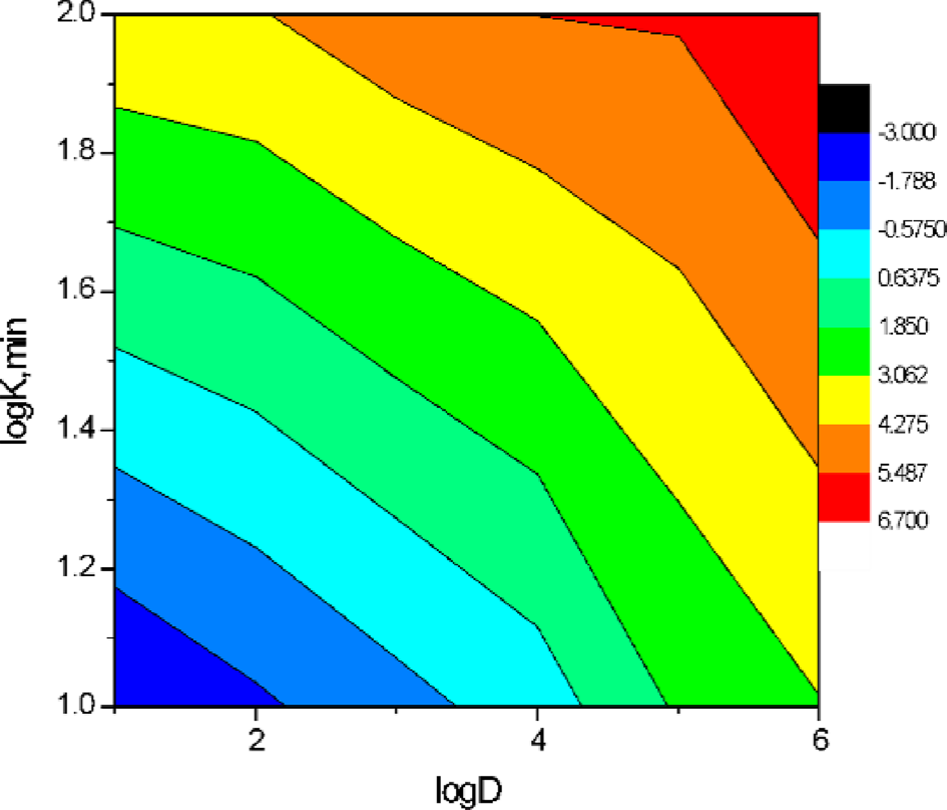
Contour plot for the effect of [Piperazine] as stripping phase on the log(K), min^−1^ and the yield of diffusion of Co(II) log yield fraction at pH > 7, [Co^2+^] = 3.4 × 10^–3^ mol/L, [D_2_EHPA] 3.3 mol/L and [HNO_3_] 2.5 mol/L at pH [1–12].

C_4_H_9_N_2_ is a stripping agent used in the separation of Co(II) ions in nitric acid using D_2_EHPA extract. Under acidic conditions, piperazine forms complexes containing ions, which reduces the separation efficiency. The effect of solution pH on piperazine ionization and cobalt (II) extraction using D_2_EHPA as extractant is shown in Fig. 10. pH 9.8 pka of strip 9.73 while at pH 6, pka of piperazine strip at 5.92. The ionization^55^ behavior of piperazine is influenced by the protonation or deprotonation of its nitrogen atoms. In an acidic environment, the ionization fraction increases due to more protons available for donation. In basic conditions, where protons are less available, the ionization fraction decreases, while in acidic conditions, where protons are more available, the fraction increases. Figure 11. When the concentration of piperazine is lower, there's less piperazine available in the solution. Increasing the pH, the solution becomes more basic. This implies a higher concentration of (OH^-^), log (1 − R) becomes more significant as the pH increases or another meaning, the contribution of the non-piperazine fraction to the overall behavior of the system becomes more pronounced in basic conditions. The lower the concentration of piperazine, the more pronounced the influence of log(1-R) becomes as the pH of the solution increases. This an important consideration when studying or modeling the behavior of solutions containing piperazine, especially at lower concentrations and higher pH values. Figure 12. 3D the optimal pH range for effective extraction and diffusion depends on the specific system and species characteristics. A contour plot in Fig. 13 can be used to determine the optimal piperazine concentration that maximizes the reaction rate and diffusion yield of cobalt(II) ions, which is useful for process optimization and the design of efficient extraction and diffusion systems^33,59–74^.

From equation (17) represent the rate of change of a variable “c” with respect to time “t”. The equation is given as: dc/dt = k; Where: dc/dt: This term presents the rate of change of the variable “c” with respect to time “t”, it means the concentration of “c” with respect to time. k: This is a constant that expresses the rate at which the variable “c” is varying with time. The equation describes the linear relationship between the change in the variable “c” and the passage of time “t”, with the rate of change determined by the constant “k”. This type of first-order differential equation is applied to model dynamical processes in which the amount or concentration of a variable is changing in an ongoing process with time.

The exponents (− 8.3, − 0.64, and 8.9) associated with each concentration term indicate the order of the reaction on the linear slopes for each of the concentrations shown above respectively at 30 °C.

17 $$ \frac{dc}{{dt}} = k\left[ {D_{2} {\text{EHPA}}} \right]^{ - 8.3} \left[ {{\text{HNO}}_{3} } \right]^{ - 0.64} \left[ {{\text{Piperazine}}} \right]^{8.9} $$

### Comparison between predictive model and experimental data

**Figure 14:**
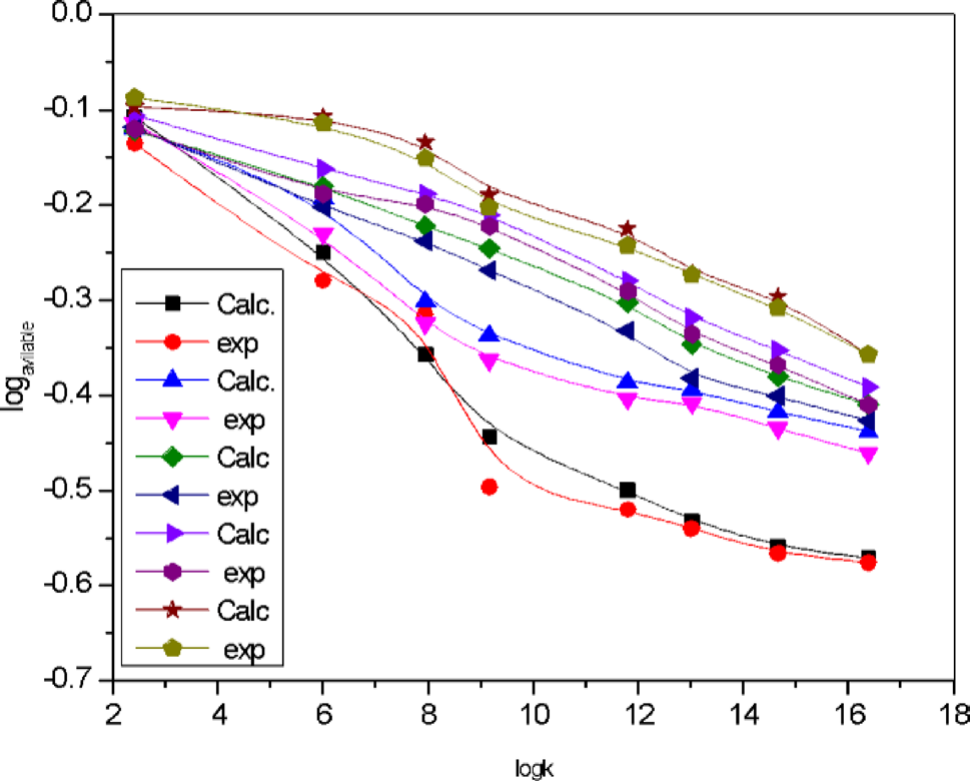
Plotting logK, min against log_variables_.

Based on theoretical principles, experimental correlations, and computational simulations, a predictive model for cobalt separation efficiency using ceramic membranes is developed. The model includes ceramic membrane properties, feed solution composition, stripping solution composition, operating conditions, and pH. From Fig. 14. The model was validated using MATLAB where it agrees with the experimental results, thus predicting a positive effect at pH values higher than 7 on the transport of Co(II) ions. However, at pH values below 7, further reduction in pH does not affect the recovery of Co(II). Mass transport in the internal phase boundary layer may be the rate controlling step of the Co(II) transfer rate. This is shown by the total combined resistance to the three effects represented by the 1/k_n,_ pKa value at pH 9.8 at the indicated concentrations. A pKa value of 9.73 represents the dissociated or dissociated form of the compound at the given pH value, which affects the transfer of Co(II) ions from aqueous solutions. This is because the effect of piperazine depends on its functional group. Transport of Co(II) ions across the ceramic membrane. The model predicted a positive effect at pH values above 7, but lower pH did not affect recovery at pH values below 7. The ionization fraction of the compound can affect the speciation and transport of Co(II) ions. The model prediction was positive at high pH, but there was no effect at pH values, indicating transport at the inner phase layer boundary. Ceramic polymer backed membranes have advantages in water treatment, mineral hydro treatment and environmental treatment. By comparing the results with the model predictions for the degree of synchronization, the results between the model predictions and experimental data can be evaluated through statistical analysis using plotting the logK, min relationship against log variables and calculating correlation coefficients. All discrepancies as well as differences between model predictions and experimental results are discussed. The discussion included simplifications or assumptions in the model, or experimental uncertainties. Or limitations in the experimental setup^71–74^.

## Conclusion

Ceramic materials are often used in extraction processes to enhance selectivity, absorption, and operational flexibility. They can improve the purity of the extracted product by selectively absorbing or binding specific compounds. Ceramic's high surface area and specific properties make it an excellent adsorbent, capturing molecules that may not interact with D_2_EHPA. They can also be refurbished, making them cost-effective and environmentally friendly. Additionally, ceramic can be used in different extraction scales, making it versatile for different applications. Combining D_2_EHPA with ceramic can produce synergistic effects, improving the overall extraction efficiency. The study revealed that optimal pH and temperature conditions are necessary for efficient Co(II) extraction in D_2_EHPA-based polymer membrane systems. D_2_EHPA Co(II) is extracted at pH below 7 and %E at 47%. Higher pH values lead to higher extraction efficiency, especially in the stripping phase, where the distribution ratio ranges from 6.5 to 9.8, which indicates better membrane partitioning. The recovery rate depends on the presence of nitrogen atoms in the piperazine or functional group of the stripping agent. The study investigates the effect of pH, piperazine concentration and membrane properties on cobalt extraction efficiency using D_2_EHPA and ceramic supported polymer membrane. The optimum pH for cobalt(II) separation is 9–11, with a higher pH favorably affecting transfer. The rate-determining step is the border mass transfer, and the pKa value indicates a slight lower than the pH of the stripping phase, which plays a role in extracting cobalt ions at a higher percentage from the extract depending on the respective functional group.

### Supplementary Information

Supplementary Information.

## Data Availability

All data generated or analyzed during this study are included in this published article [and its supplementary information files].
