# Supplementary material for: Predictive modeling of pH on the transport of Co(II) Ions from aqueous solutions through supported ceramic polymer membrane
Source: Sci Rep. 2024 Jun 26;14:14778. doi: 10.1038/s41598-024-63854-7 (PMC11208598; doi:10.1038/s41598-024-63854-7)
Supplement: Supplementary file 1 — Supplementary Information. [file 41598_2024_63854_MOESM1_ESM.docx]

*C_f_*

*Particle flux*

*B_f_*

*C_bd_*

*Concentration of particle back-diffusion*

*αJC_m_*

[(C_14_H_24_N_2_O_9_) /diluent

HNO_3_

^3+^NO

*Permeate*

*JC_p_*

*C_p_*

*C_m_*

Fig.S1. Technicality, of Co (II) ions transport via Support Ceramic Polymer Membrane (SCPM)


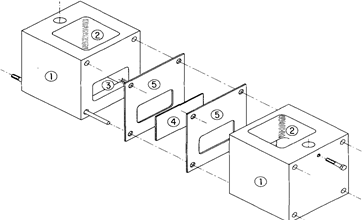

Fig.S2.Scheme for pertraction apparatus .(1) Double shield glass outer vessel;(2)Teflon cross-stirring blade;(3) Supported membrane(cellulose nitrate);(4)feed compartment;(5)strip compartment

*Si(OR)_4_+4H_2_O Si(OH)_4_+4ROH*

*Undergo-gelation*

Drying &Washing

TEOS+DMF

Si(OH)_4_ SiO_2_+2H_2_O

*Silica-gel*

*Modification*


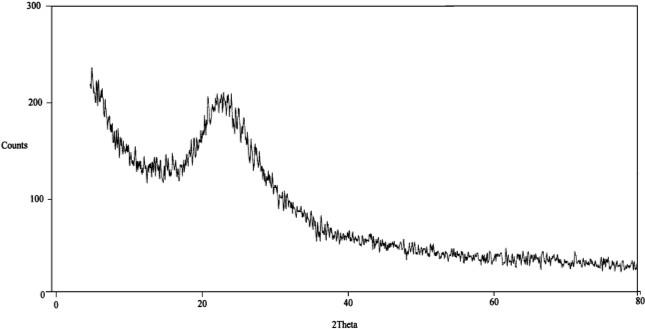


*Silica particles*

Fig.S3. Flow chart for preparing of Ceramic polymer supported membrane (CPSM)

*Fig.5. Effect of pH of the Co (II) % E from [(C_4_H_10_N_2_)]~0.48M , [HNO_3_]=2.5 mol/L at [Co^2+^] = 0.8-4x10^-3^ mol/L, [HNO_3_]~2.5 mol/L at pH [9.8]. [D_2_EHPA]~ 3.3 mol/L*

*Fig.4. Effect of pH on the extraction percentage of cobalt ion (II) on the extractant using D_2_EHPA, [Co^2+^] = 0.8-4x10^-3^ mol/L, [(C_4_H_10_N_2_)]~0.48 M, [D_2_EHPA]~ 3.3 mol/L, [HNO_3_]~2.5 mol/L at pH= [0-5].*

*Fig.6. Effect of pH on the temperatures in [D_2_EHPA]~ 3.3 mol/L, [HNO_3_]~2.5 mol/L at pH= [1-4.5].*

*Fig.7. Effect of pH on the temperatures in [(C_4_H_10_N_2_)]~0.48M] =] ~3x10^-2^ mol/L,pH=[9.8*]

*Fig.8.Effect of Co (II) ion concentration on the flux (J)*

*Fig.9. Effect of HNO_3_ concentration on flux of cobalt ion [Co^2+^] 3.4x10^-3^ mol/L, [Piperazine] 0.48 M, [D_2_EHPA] 3.3 mol/L and [HNO_3_] 0.5-2.2mol/L at pH 9.8 at 30^0^ C.*

*Fig10. Influence of pH of Piperazine strip ionization and fraction of extraction of [Co^2+^] = 3.4x10^-3^ mol/L, [D_2_EHPA] 3.3 mol/L and [HNO_3_] 2.5 mol/L at pH [1-11]*

*Fig.11. Modeling pH of Piperazine stripping phase 0.45M on Co (II) ion concentration diffusion with CPSM with carrier D_2_EHPA in kerosene.[Co^2+^]= 3.4x10^-3^ mol/L, L, [D_2_EHPA] 3.3 mol/L and [HNO_3_] 2.5 mol/L at pH [0-12]*

*Fig.12. 3D for effect of [piperazine] 0.45M as stripping phase at pH [1-12] on the rate of reaction K,_min_ and the yield of diffusion of Co(II) ion concentration,[Co^2+^]= 3.4x10^-3^ mol/L, mol/L, [D_2_EHPA] 3.3 mol/L and [HNO_3_] 2.5 mol/L .*
